# ASPirin Intervention for the REDuction of colorectal cancer risk (ASPIRED): a study protocol for a randomized controlled trial

**DOI:** 10.1186/s13063-016-1744-z

**Published:** 2017-02-01

**Authors:** David A. Drew, Samantha M. Chin, Katherine K. Gilpin, Melanie Parziale, Emily Pond, Madeline M. Schuck, Kathleen Stewart, Meaghan Flagg, Crystal A. Rawlings, Vadim Backman, Peter J. Carolan, Daniel C. Chung, Francis P. Colizzo, Matthew Freedman, Manish Gala, John J. Garber, Curtis Huttenhower, Dmitriy Kedrin, Hamed Khalili, Douglas S. Kwon, Sanford D. Markowitz, Ginger L. Milne, Norman S. Nishioka, James M. Richter, Hemant K. Roy, Kyle Staller, Molin Wang, Andrew T. Chan

**Affiliations:** 1 Clinical and Translational Epidemiology Unit, Department of Medicine, Massachusetts General Hospital and Harvard Medical School, Boston, MA USA; 2 Division of Gastroenterology, Department of Medicine, Massachusetts General Hospital and Harvard Medical School, Boston, MA USA; 3 The Ragon Institute of MGH, MIT and Harvard, Cambridge, MA USA; 4 McCormick School of Engineering, Northwestern University, Evanston, IL USA; 5 Broad Institute, Cambridge, MA USA; 6 Department of Biostatistics, Harvard T.H. Chan School of Public Health, Boston, MA USA; 7 Department of Epidemiology, Harvard T.H. Chan School of Public Health, Boston, MA USA; 8 Case Western Reserve University School of Medicine and University Hospitals of Cleveland, Cleveland, OH USA; 9 Eicosanoid Core Laboratory, Division of Clinical Pharmacology, Vanderbilt University, Nashville, TN USA; 10 Section of Gastroenterology, Boston Medical Center, Boston, MA USA; 11 Channing Division of Network Medicine, Department of Medicine, Brigham and Women’s Hospital and Harvard Medical School, Boston, MA USA; 12 Division of Gastroenterology and Clinical and Translational Epidemiology Unit, Massachusetts General Hospital, GRJ-825C, Boston, MA 02114 USA

**Keywords:** Aspirin, Colorectal cancer, Biomarker, Chemoprevention

## Abstract

**Background:**

Although aspirin is recommended for the prevention of colorectal cancer, the specific individuals for whom the benefits outweigh the risks are not clearly defined. Moreover, the precise mechanisms by which aspirin reduces the risk of cancer are unclear. We recently launched the ASPirin Intervention for the REDuction of colorectal cancer risk (ASPIRED) trial to address these uncertainties.

**Methods/design:**

ASPIRED is a prospective, double-blind, multidose, placebo-controlled, biomarker clinical trial of aspirin use in individuals previously diagnosed with colorectal adenoma. Individuals (*n* = 180) will be randomized in a 1:1:1 ratio to low-dose (81 mg/day) or standard-dose (325 mg/day) aspirin or placebo. At two study visits, participants will provide lifestyle, dietary and biometric data in addition to urine, saliva and blood specimens. Stool, grossly normal colorectal mucosal biopsies and cytology brushings will be collected during a flexible sigmoidoscopy without bowel preparation. The study will examine the effect of aspirin on urinary prostaglandin metabolites (PGE-M; primary endpoint), plasma inflammatory markers (macrophage inhibitory cytokine-1 (MIC-1)), colonic expression of transcription factor binding (transcription factor 7-like 2 (TCF7L2)), colonocyte gene expression, including hydroxyprostaglandin dehydrogenase 15-(NAD) (*HPGD*) and those that encode Wnt signaling proteins, colonic cellular nanocytology and oral and gut microbial composition and function.

**Discussion:**

Aspirin may prevent colorectal cancer through multiple, interrelated mechanisms. The ASPIRED trial will scrutinize these pathways and investigate putative mechanistically based risk-stratification biomarkers.

**Trial registration:**

This protocol is registered with the U.S. National Institutes of Health trial registry, ClinicalTrials.gov, under the identifier NCT02394769. Registered on 16 March 2015.

**Electronic supplementary material:**

The online version of this article (doi:10.1186/s13063-016-1744-z) contains supplementary material, which is available to authorized users.

## Background

### Background and rationale

Colorectal cancer (CRC) remains the second leading cause of cancer-related deaths in the United States despite the increasing adoption of screening [1]. Thus, the development of alternative strategies for prevention remains a high priority. Aspirin (acetylsalicylic acid) has emerged as perhaps the most promising chemopreventive agent to date with numerous basic, epidemiological and clinical studies supporting its anticancer effects, especially against CRC [2]. In view of this compelling evidence, the United States Preventive Services Task Force recently recommended the use of aspirin for the primary prevention of CRC as well as cardiovascular disease (CVD) in individuals aged 50–69 years with elevated cardiovascular risk profiles [3].

Nonetheless, aspirin is associated with harms, including gastrointestinal bleeding, for which the risk increases with age, as well as dose and duration of use [2, 3]. The field is now at a critical juncture where broader adoption of an aspirin-based chemoprevention strategy requires human data clarifying the optimal dose and mode of action, which ultimately may help identify subgroups of individuals for whom the preventive benefits outweigh the harms. Carefully designed randomized clinical trials that examine a range of molecular biomarkers associated with aspirin’s mechanisms will provide a cost-efficient means to illuminate the molecular underpinnings of aspirin’s chemopreventive effects and validate putative risk-stratification biomarkers. We have recently initiated the “ASPirin Intervention for the REDuction of colorectal cancer risk” (ASPIRED) trial to achieve this goal.

### Objectives

Within the gastroenterology practice of Massachusetts General Hospital (MGH), we will conduct a prospective, double-blind, placebo-controlled, randomized clinical trial to measure the effects of daily low-dose (81 mg/day) and standard-dose (325 mg/day) aspirin on urine, plasma, stool, and tissue biomarkers associated with CRC among patients with a prior adenoma history. This study is designed to advance the mechanistic understanding of aspirin’s anticancer mode of action with the goal of identifying individuals most likely to benefit from an aspirin-based chemoprevention regimen. Specifically, we hypothesize that:

- Aspirin at 81 mg/day or 325 mg/day reduces urinary prostaglandin metabolites (PGE-M), a biomarker of prostaglandin tone, to levels associated with low risk of colorectal neoplasia
- Aspirin at 81 mg/day or 325 mg/day reduces plasma macrophage inhibitory cytokine-1 (MIC-1), an inflammatory biomarker
- Aspirin at 81 mg/day or 325 mg/day impairs binding of transcription factor 7-like 2 (TCF7L2)/T-cell factor 4 (TCF4) at the *8q24* CRC risk locus in colonic epithelium, particularly among individuals with the *8q24* CRC susceptibility allele
- Aspirin at 81 mg/day or 325 mg/day lowers expression of *Wnt* signaling genes (*CTNNB1*, *AXIN-2* and *MYC*) and hydroxyprostaglandin dehydrogenase 15-(NAD) (*HPGD*) in colonic epithelium
- Aspirin at 81 mg/day or 325 mg/day is associated with less neoplastic nanomorphological cellular signatures from brushings of normal colorectal mucosa
- Aspirin at 81 mg/day or 325 mg/day is associated with an increase in oral and gut microbial diversity, function, and genomic richness and inhibits microbial pathways associated with CRC

## Methods/design

### Trial design

ASPIRED is a prospective, double-blind, placebo-controlled, three-arm randomized controlled trial. We will target 180 individuals (60 per arm) for enrollment.

### Participants, interventions and outcomes

#### Study setting

A single-center, academic hospital (MGH)

#### Eligibility criteria

Potential participants will be identified within the gastroenterology practice of MGH. Eligible adults (aged 18–80 years) will have had at least one adenoma removed during a qualifying endoscopy within 9 months of study enrollment, confirmed via official pathology reports. Full inclusion and exclusion criteria are included in Table 1.

**Table 1:** ASPIRED eligibility criteria

Inclusion criteria	Exclusion criteria
• Adenoma removed during qualifying endoscopy (as confirmed by pathology) • Not currently taking aspirin (any dose) within the last 6 months • Age 18–80 years • Able to swallow pills • Ability to understand and the willingness to sign a written Informed Consent Document • ECOG performance status ≤2 • (Karnofsky Index score ≥60%)	• Any adenoma that was not completely removed during previous colonoscopy • Known diagnosis of familial adenomatous polyposis (FAP) or hereditary non-polyposis colorectal cancer (HNPCC, Lynch syndrome) • Diagnosis of inflammatory bowel disease, liver or kidney disease, or bleeding diathesis • Any prior diagnosis of gastrointestinal cancer (including esophageal, small intestinal, colon, pancreatic), or any diagnosis of other cancers (with the exception of non-melanomatous neoplasia of skin) in which there has been any active treatment within the last 3 years • Use of any nonaspirin, nonsteroidal anti-inflammatory drug (NSAID) at any dose at least 3 times/week during the 2 months prior to randomization • History of aspirin intolerance, bleeding diathesis, peptic ulcer or gastrointestinal bleed, endoscopic complications, or contraindication to colonoscopy • History of allergic reactions attributed to compounds of similar chemical or biologic composition to aspirin • Taking any anticoagulant agent (e.g., warfarin) or antiplatelet agent (e.g., clopidogrel) • Receiving any other investigational agents • Uncontrolled intercurrent illness including, but not limited to, ongoing or active infection, symptomatic congestive heart failure, unstable angina pectoris, cardiac arrhythmia, or psychiatric illness/social situations that would limit compliance with study requirements • Pregnant or breastfeeding

*ECOG*, Eastern Cooperative Oncology Group

#### Interventions

The first dose of the study medication will be provided to participants immediately upon completing the initial baseline flexible sigmoidoscopy (start of randomization). Aspirin is an odorless, white, needle-like crystalline or powdery substance. Generic aspirin is provided as an oral tablet at an 81-mg or a 325-mg dose. To maintain blinding, we use specially designed formulations in which aspirin tablets are crushed into a powder. An amount of powder that reflects the appropriate dose is then placed in a gel capsule with a lactose filler. In the case of placebo, an identically sized capsule filled only with lactose is used. Participants will be instructed to consume one study capsule per day with food and a full glass of water until they return for their final visit. The final visit will occur a minimum of 8 weeks, but no more than 12 weeks after the initial visit. The study duration is designed to maximize feasibility and compliance. Moreover, in a pilot study, two doses of 650 mg of aspirin, 14 h apart, were sufficient to reduce urinary PGE-M levels by 44% [4]. Furthermore, aspirin and other NSAID agents have been shown previously to have an effect on recurrent adenoma risk within as little as 1 year, [5, 6] which suggests that biological impact of the treatment on colonic epithelium may be reasonably measured within 2–3 months. The capsules will be packaged in a pill bottle containing 84 capsules (12-week daily supply) and participants will return any unused capsules and the bottle to study staff at the final visit. Remaining capsules will be counted as a measure of compliance. Weekly calls from study staff during this 8–12-week period will be used to monitor adherence and adverse events and promote retention. Any use of nonstudy aspirin or other NSAID during this time period will result in the participant being withdrawn from the study and an exit visit will be performed at that time. Participants will be provided with US$200 compensation and free parking for the baseline visit and US$300 and free parking for the final visit, resulting in a total of US$500 following successful completion of the study.

#### Outcomes

This trial aims to examine the post-treatment effect of low-dose (81 mg/day) and standard-dose (325 mg/day) aspirin on pretreatment urinary PGE-M. Secondary endpoints include the effects of aspirin on the following CRC-associated biomarkers:

- Plasma MIC-1, an inflammatory biomarker
- TCF7L2/TCF4 binding at the *8q24* CRC risk locus in colonic epithelium
- Wnt signaling proteins (i.e., β-catenin, AXIN-2 and MYC) and *HPGD* gene expression as measured by ribonucleic acid (RNA)-sequencing (RNA-seq) of sorted colonic epithelial cell populations
- Spectral biomarkers of colorectal carcinogenesis from cytology brushings
- Bacterial populations and products associated with CRC in saliva and stool

A detailed description of the rationale for the selection of these biomarkers and their biological relevance to aspirin chemoprevention appears in the “Discussion” section.

#### Participant timeline

**Fig. 1:**
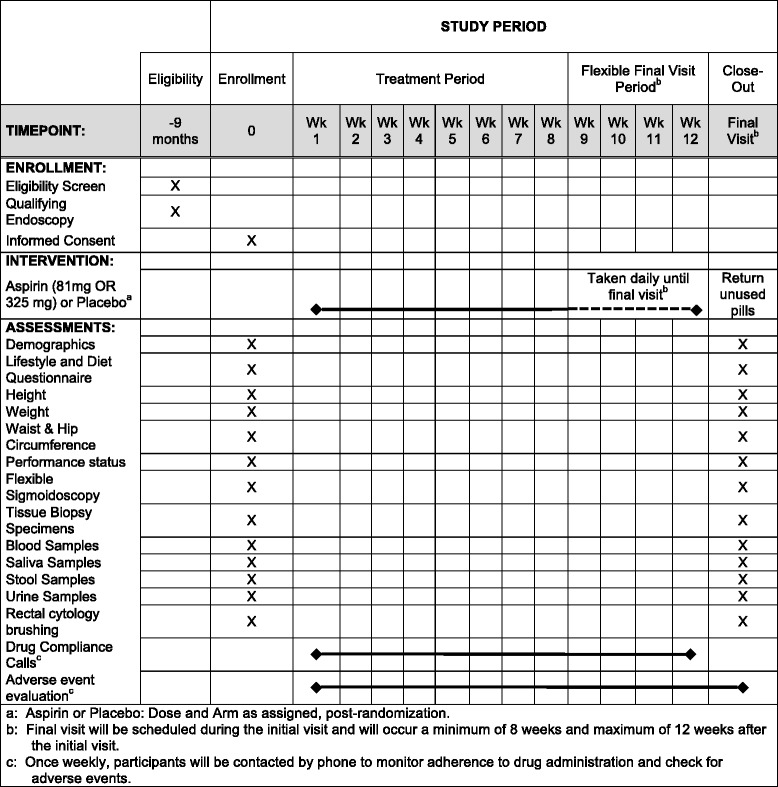
ASPIRED study timeline (Standard Protocol Items: Recommendations for Interventional Trials (SPIRIT) diagram)

**Fig. 2:**
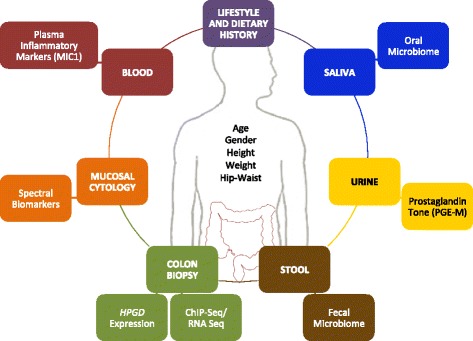
ASPIRED assessments and biospecimens. Overview of patient assessments and biospecimens collected in the ASPIRED trial for correlative science experiments. Participants are asked to provide relevant lifestyle and dietary histories via paper questionnaire. Anthropometric measurements are collected at each visit by study staff. In addition, participants provide urine, saliva, blood, stool, and tissue samples at both study visits

The study timeline (Standard Protocol Items: Recommendations for Interventional Trials (SPIRIT) diagram consistent with the SPIRIT Checklist. The SPIRIT Checklist is attached as Additional file 1: SPIRIT Checklist) appears as Fig. 1. At the initial (baseline) visit, the study physician obtains written informed consent for the study, as well as a standard clinical consent for a flexible sigmoidoscopy. Following completion of this visit, the participants are considered “on treatment” (start of randomization) and take blinded aspirin or placebo daily until the end of the treatment period (8–12 weeks later). At the end of the treatment period, participants will return for a final visit during which a second flexible sigmoidoscopy will be performed. Both clinical visits are identical in their data and specimen collection approaches. Participants are asked to complete a brief lifestyle and dietary questionnaire, which is subsequently transferred (via double entry) to a secure REDCap electronic database system. Trained study staff will collect measurements of height, weight, waist and hip circumference, as well as blood (three 10-mL vials), urine (approximately 20 mL) and saliva specimens (2–3 mL). Twenty milliliters of whole blood will be immediately aliquoted and frozen at −80 °C. Ten milliliters of whole blood will be immediately centrifuged for serum and buffy coat collection, aliquoted and frozen at −80 °C. Saliva is immediately aliquoted, frozen on dry ice, and stored at -80 °C. Urine is immediately stored on wet ice, aliquoted within 1 h and frozen at −80 °C. A study gastroenterologist will then perform a flexible sigmoidoscopy, advancing to the level of the distal sigmoid colon. No bowel preparation will be necessary for the procedure. Thus, stool will be either aspirated through the endoscope or collected using a Roth net then aliquoted into cryovials, immediately frozen on dry ice, and stored at -80 °C. The gastroenterologist then identifies an area of the rectosigmoid junction, clear of any stool, to collect cellular material using an endoscopic cytology brush. The brush heads are cut and placed immediately into a tube containing 25% ethanol, then placed on wet ice until storage at 4 °C. These samples are kept cold through shipment and processed for partial wave spectroscopy (PWS) within 2 − 3 days of collection. Last, the gastroenterologist collects 24 pinch-biopsy specimens that are processed and stored under multiple conditions (e.g., formalin-fixed, paraffin-embedded; flash-frozen; culture media; etc.) for future assays. A summary of the patient assessments and biospecimens collected appears as Fig. 2.

#### Sample size

We calculated the sample size required to estimate the effect of aspirin on our primary endpoint, urinary PGE-M, using the two aspirin groups combined versus placebo. The null hypothesis is: *H*_0_: *Δ*_aspirin_ – *Δ*_placebo_ = 0 versus *H*_*A*_: *Δ*_aspirin_ – *Δ*_placebo_ ≠ 0, where *Δ*_aspirin_ and *Δ*_placebo_ are the mean changes in urinary PGE-M level from baseline to end of treatment for the intervention and placebo groups, respectively. Based on prior studies [4, 7, 8] we assumed a standard deviation (SD) of 5.0 for a single measurement of PGE-M and an intraclass correlation (ICC) of 0.1. We will recruit 60 participants in each group to account for the possibility of dropout (20%). Assuming 45 participants in the placebo group and 90 participants in the combined aspirin group will complete the study, we estimate 90% power to detect a mean change of PGE-M level in the aspirin group of 4.0 ng/mg, compared with no change in the placebo group, assuming a type I error rate of 0.05. This minimum detectable difference in mean change is consistent with the difference in the median level of PGE-M among individuals at high risk for adenoma compared to those with low risk [7].

#### Recruitment

Patients who meet the inclusion criteria will be identified through investigators during their routine clinical practice, supplemented by a periodic query of the MGH endoscopy and pathology databases. Potentially eligible participants are approached by letter from their treating physician. Two weeks after receiving the letter, study staff will contact eligible parties and screen for eligibility via phone interview. If eligibility is established, individuals are mailed a copy of the Informed Consent Documents (Informed Consent Documents included as Additional file 2). Written informed consent is obtained from each participant by a study physician prior to performing any study-related procedures.

### Assignment of interventions

#### Allocation

The randomization schedule will not be disclosed to the investigator or any personnel involved in the conduct of the study before the database is locked, except as described here. Enrollment and randomization will be carried out by the Office of Data Quality within the Dana-Farber/Harvard Cancer Center (DF/HCC) into three arms: placebo, 81 mg/day, or 325 mg/day. Randomization assignment is dispatched directly to the MGH Research Pharmacy by the Office of Data Quality.

#### Blinding

Neither the participant nor the study physician or any study personnel will know which of the three treatments (placebo, 81 mg/day, or 325 mg/day) the participant is receiving. The Office of Data Quality will provide randomization assignment to the MGH Research Pharmacy. The MGH Research Pharmacy will provide blinded capsules. The investigator or treating physician may unblind a participant’s treatment assignment only in the case of emergency, when knowledge of the study treatment is essential for the appropriate clinical management or welfare of the participant. If the blind is broken by the investigator, the participant will be permanently discontinued from the study and an early termination assessment will be completed. If unblinding is necessary, study staff will contact the Office of Data Quality.

### Data collection, management and analysis

#### Data management

Upon consent, participants will be assigned a three-digit unique identifier assigned sequentially by study staff. All data and specimens collected will be stored and labeled using this coded identifier. Data collected via questionnaires and clinical research forms will be transferred via double entry to a secure REDCap electronic database system.

#### Statistical methods

The primary and secondary efficacy analyses will be performed on an intention-to-treat basis, with endpoints determined for all patients who complete the final flexible sigmoidoscopy and sample collection, regardless of whether the patient complied with study drug use. To illustrate the statistical plan, we provide the analysis plan intended for the primary endpoint. The primary endpoint will utilize an intention-to-treat analysis comparing the effect of each treatment on end-of-treatment change in urinary PGE-M compared to the change in the placebo group, using a two-sample *t* test. In secondary analyses, a multivariate linear regression model adjusting for other covariates will be used in case there exist imbalances in determinants of change in PGE-M levels between arms. A robust variance estimate will be used to eliminate any normality assumptions for the residuals. Statistical methods for each secondary endpoint are available upon request to the authors.

### Monitoring

#### Data monitoring

The DF/HCC Data and Safety Monitoring Committee (DSMC) will review and monitor toxicity and accrual data from this study. The DSMC is composed of clinical specialists with experience in oncology and who have no direct relationship with the study. Information that raises any questions about participant safety will be addressed with the overall principal investigator and study team. The DSMC will review each protocol up to four times a year, or more often if required, to review toxicity and accrual data. Information to be provided to the committee may include: up-to-date participant accrual; current dose-level information; all grade 2 or higher unexpected adverse events that have been reported; any response information; audit results, and a summary provided by the study team. Other information (e.g., scans, laboratory values) will be provided upon request.

### Ethics and dissemination

#### Research ethics approval

This study and its respective consenting procedures was approved by the DF/HCC Institutional Review Board (Protocol # 14-496) on 9 December 2014 and activated on 10 April 2015. The first participant was enrolled on 6 July 2015. The current protocol (Amendment 10/Version 12) was approved via continuing annual review on 24 October 2016.

#### Protocol amendments

All amendments are reviewed by the DF/HCC Institutional Review Board and communicated as necessary in writing to relevant parties.

#### Consent

Written, signed, informed consent will be collected by a study physician prior to any study-related procedures.

#### Confidentiality

We will take measures to protect the privacy and security of all participants’ personal information, but we cannot guarantee complete confidentiality of study data. Medical information created by this research study may become part of a DF/HCC research database. The results of this research study may be published, but participants will not be identified without their permission.

#### Declaration of interests

ATC previously served as a consultant for Bayer Healthcare, Millennium Pharmaceuticals, Aralaz Pharmaceuticals, and Pfizer Inc. HKR is a minority shareholder of Nanocytomics LLC and American BioOptics. SM has an unlicensed intellectual property patent covering use of *HPGD* to predict NSAID response. This study was not funded by Bayer Healthcare, Millennium Pharmaceuticals, Aralaz Pharmaceuticals, Pfizer Inc., Nanycytomics LLC, or American BioOptics. No other competing interests exist. The other authors declare that they have no competing interests.

#### Access to data

Coded samples and/or data may be sent by MGH or DF/HCC to other researchers who are also studying aspirin chemoprevention and/or collaborating with the study team including, but not limited to, the National Institutes of Health, the Ragon Institute, the Dana-Farber Cancer Institute, Children’s Hospital, Vanderbilt University, the Broad Institute, Northwestern University, and the Harvard T.H. Chan School of Public Health. All other scientists and/or collaborators must meet MGH requirements for sharing samples and/or data including treating the data or materials as medically confidential, obtaining approval from their Human Subjects Review Boards, and agreeing not to share the data or materials with other parties. Affiliated researchers or laboratories outside of DF/HCC and MGH will never know who a participant is, nor have access to the code linking the samples back to the participants.

#### Dissemination policy

The results should be made public within 24 months of reaching the end of the study. The end of the study is the time point at which the last data items are to be reported, or after the outcome of data are sufficiently mature for analysis as defined in the statistical analysis section. We plan to publish in a peer-reviewed journal; thus, the initial release may be an abstract that meets the requirements of the International Committee of Medical Journal Editors. A full report of the outcomes should be made public no later than 3 years after the end of the study. Results will also be available through ClinicalTrials.gov.

## Discussion

We hypothesize that aspirin, by reducing the risk of multiple cancers and CVD, has a favorable risk-benefit profile for most individuals and influences several neoplastic pathways which can be exploited as biomarkers of chemopreventive efficacy. The following study will examine dose-dependent effects of aspirin treatment on specific biomarkers of colorectal carcinogenesis. In doing so, we aim to provide causality for aspirin’s overall risk-benefit established by other studies. We and others have put forth considerable effort to determine measurable biomarkers implicated in colorectal carcinogenesis, which we have recently summarized in a comprehensive review [2]. A brief discussion of the biomarkers that will be measured during ASPIRED and their significance is provided below:

### Urinary PGE-M (primary endpoint)

We have previously shown that aspirin’s influence on CRC is mediated at least in part through inhibition of prostaglandin-endoperoxide synthase-2 (PTGS-2; or cyclooxygenase (COX)-2), [9, 10] which catalyzes production of prostaglandin E2 (PGE_2_), leading to induction of proliferation, migration and invasiveness, promotion of angiogenesis, resistance to apoptosis and modulation of cellular and humoral immunity within the tumor. We have estimated overall prostaglandin tone by measuring its major metabolite, PGE-M (11-alpha-hydroxy, 9, 15-dioxo-2, 3, 4, 5-tetranor-prostane-1, 20-dioic acid), in urine [8]. This assay is widely accepted as the most valid method of quantifying systemic PGE_2_ production in vivo [11]. Prediagnostic levels of PGE-M are associated with risk of CRC and adenoma [12–14]. In the Nurses’ Health Study, women in the highest quartile of PGE-M levels had a multivariate odds ratio (OR) of 1.66 (CI, 1.04–2.66) for high-risk adenoma compared to women in the lowest [7]. Moreover, aspirin/NSAIDs was associated with a significant reduction in adenoma risk among women with high (OR, 0.61; CI, 0.43–0.87) but not low PGE-M (OR 1.05; CI, 0.50–2.19). These results support the potential for PGE-M to define subsets of the population who may obtain differential chemopreventive benefit from aspirin. PGE-M has also been associated with gastric and breast cancer [15–17]. Moreover, in a study of 10 individuals, two doses of 650 mg of aspirin administered 14 h apart reduced PGE-M by 44% [4]. Thus, PGE-M may also serve as a biomarker to assess the effectiveness of aspirin in reducing the risk of adenoma. However, randomized studies are needed to determine if aspirin at typical doses of either 81 mg/day or 325 mg/day inhibits generation of PGE-M to a level associated with low adenoma risk.

### Plasma MIC-1

The circulating inflammatory cytokine MIC-1 (also known as growth differentiation factor 15 (GDF-15), [18] placental bone morphogenetic protein (PLAB), [19] or prostate-derived factor (PDF) [20]) may be an important mediator in systemic inflammatory response [21, 22]. MIC-1 has also been linked to cancers, including those of the prostate, thyroid, pancreas and colon, [23, 24] and recurrent adenoma [25]. Experimental evidence suggests that MIC-1, as a member of the human transforming growth factor-β (TGFβ1) superfamily, may play a specific role in carcinogenesis [26–28]. We recently reported that the multivariable relative risk (RR) for CRC was 1.93 (CI, 1.27–2.94) comparing extreme quintiles of MIC-1 (P_*trend*_ = 0.004). Among individuals with high MIC-1, aspirin/NSAIDs were associated with a lower risk of PTGS-2-positive (multivariate RR = 0.60; CI, 0.41–0.88) but not PTGS-2-negative CRC (multivariate RR = 1.21; CI, 0.71–2.07) [29]. Taken together, these results support the potential for plasma MIC-1 to serve as a biomarker to define subsets of the population who may obtain differential chemopreventive benefit from aspirin. Thus, randomized studies are needed to determine whether aspirin (81 mg/day or 325 mg/day) specifically reduces levels of MIC-1.

### ChIP-seq in colonic epithelium, Wnt/β-catenin

Activation of the Wnt/β-catenin signaling pathway plays a critical role in colon tumorigenesis [30–32]. β-catenin is a key effector in Wnt signaling since T-cell factor family members transcribe their target genes only when bound to β-catenin. Several studies have shown that aspirin may directly suppress Wnt signaling through PTGS-independent pathways [33–36]. In addition, compelling evidence supports a critical interaction between prostaglandin pathways and Wnt signaling such that aspirin may inhibit Wnt signaling through suppression of PTGS-mediated synthesis of PGE_2_ [37–42]. Although these experimental data are compelling, human studies in support of an effect of aspirin mediated through Wnt are limited. In a case-control study of 76 patients, aspirin or ibuprofen use was associated with decreased nuclear staining of β-catenin and the Wnt target gene *C﻿CND1* (﻿*cyclin D1﻿﻿﻿﻿*) in sporadic adenoma [35]. Consistent with these findings, we found that the benefit of regular aspirin use on CRC risk was most pronounced in individuals with T alleles of rs6983267, [43] an *8q24* CRC susceptibility single nucleotide polymorphism [44–46] that we have shown is associated with impaired β-catenin binding to TCF4 adjacent to *MYC* [47]. In a murine model, rs6983267 influences *MYC* expression and intestinal tumorigenesis [48]. We corroborated these results by ChIP-seq showing that aspirin influenced binding of TCF4 in CRC cell lines heterozygous for rs6983267 [43]. The next important step will be to determine if, in vivo, aspirin results in differential binding of TCF4 in regulatory sites adjacent to key cancer-associated genes such as *8q24* within colonic epithelium.

### Gene expression in colonic epithelium – *HPGD* and *Wnt* signaling

The synthesis of tumor-promoting prostaglandins is regulated not only by PTGS-2, but also by the PGE_2_-catabolizing enzyme HPGD, which acts as a physiological antagonist to PTGS-2 [49, 50]. *HPGD* is highly expressed in normal colon and is ubiquitously downregulated in CRC [51–56]. In the Nurses’ Health Study and the Health Professionals’ Follow-up Study we demonstrated that *HPGD* expression may be used as a risk-stratification biomarker [57]. Using a validated RT-qPCR assay to quantify *HPGD* messenger ribonucleic acid (mRNA) expression in normal colonic mucosa, [58] we found that the multivariate hazard ratios associated with aspirin use were 0.49 (CI, 0.34–0.71) among those with high *HPGD* within normal colon but 0.90 (CI, 0.63–1.27) among subjects with low expression of *HPGD* (*p*_heterogeneity_ = 0.02). These results suggest that the anticancer activity of aspirin in colonic mucosa is dependent on high *HPGD* expression, with low levels of *HPGD* expression conferring resistance to aspirin’s tumor-preventive effects. Despite these findings, however, it is unclear if aspirin directly alters *HPGD* levels. A prior study showed that β-catenin/TCF4 binds the *HPGD* promoter to downregulate *HPGD* expression [59]. This would suggest that if aspirin treatment functions through inhibition of β-catenin/TCF4 binding, *HPGD* expression should also be upregulated. This would lower PGE_2_ levels, potentially serving as negative feedback by further weakening β-catenin function. In a pilot study of 45 patients, we found that aspirin (325 mg/day) was associated with a 10% increase in colonic *HPGD* expression. However, the sample size was too small to determine statistical significance (*p* = 0.12) [58]. Thus, a larger randomized treatment study is needed to determine if aspirin (81 mg/day or 325 mg/day) specifically inhibits gene expression in colonic cells associated with the Wnt signaling pathway (*CTNNB1*, *AXIN-2* and *MYC)* and *HPGD.*

### Nanomorphological alterations in colorectal cancer

Cell nanoscale architecture (e.g., fundamental cellular building blocks such as ribosomes, nucleosomes, etc.) is inherently linked to biochemical and genomic processes [60–62]. Nanoscale alterations to chromatin’s structure are one of the earliest events in carcinogenesis and a common denominator of multiple molecular pathways. These morphological alterations result in the appearance of “gold-standard” histopathological markers of dysplasia and neoplasia across most cancer types including CRC. In comparison to the micromorphological markers of dysplasia and malignancy, nanomorphological events occur at an earlier stage of carcinogenesis. In order to perform nanocytology analyses on rectal swabs obtained from the participants of the study, we will use PWS microscopy, a technique which couples spectroscopy with microscopy [63]. Recently, PWS-based nanocytology measurements were used as the primary endpoint for a randomized, placebo-controlled trial of aspirin [64]. Although the results were not statistically significant, the results suggested that aspirin use was associated with less neoplastic signatures from rectal brushings of normal mucosa. The ASPIRED trial plans to enroll more than twice the number of participants of this initial trial and may be powered to gain valuable insight into the earliest measurable benefits of aspirin chemoprevention.

### Aspirin, the oral and gut microbiome, and colorectal cancer

The oral and gut microbiomes play critical roles in epithelial cell proliferation and differentiation, intestinal immunity, nutrient processing and metabolite production, and resistance to infection by pathogenic organisms [65, 66]. Moreover, growing evidence has linked specific changes in gut microbial communities with CRC [67]. For example, cross-sectional studies have shown an increased abundance of immunomodulatory and tumor-permissive genera such as *Fusobacterium*, *Enterobacteriaceae* and *Porphyromonas* and a depletion of microbes that may protect against tumorigenesis, including *Firmicutes*, *Slackia*, *Roseburia*, *Faecalibacterium*, *Eubacterium rectale*, nonenterotoxigenic *Bacteroides* and *Clostridia* [67–69]. It remains unclear, however, how these bacteria mechanistically influence carcinogenesis. Animal and in vitro studies have provided some evidence that certain bacterial communities promote a proinflammatory environment, leading to the induction of PTGS-2, [70–72] thereby implicating a role for the microbiome in mediating the chemopreventive properties of aspirin. To date, no studies have determined the biomolecular mechanisms by which oral or gut microbial activity implicated in CRC risk and progression may be altered or respond to aspirin treatment. Defining specific mechanisms by which the oral and gut microbiota mediates the effect of aspirin on CRC will provide compelling evidence of aspirin’s chemopreventive properties and strengthen the microbiome-CRC relationship. Moreover, these studies may lead to novel strategies for CRC prevention.

In summary, the ASPIRED trial is designed to provide validation of, and insight into, the many proposed chemoprevention mechanisms of aspirin. In addition, these pathways may inform clinical practice by providing actionable risk-stratification biomarkers. Importantly, the trial has been designed with a forward-looking perspective where biobanked specimens have been stored in multiple conditions to allow flexibility and accommodate future advances in the field. We have selected, a priori, several biomarkers as study endpoints based upon established data highlighting their promise as markers of risk-stratification for CRC and aspirin chemoprevention. Notably, each biomarker has distinct characteristics that may impact how they ultimately are adopted in the clinic. Examining circulating or metabolic factors (e.g., MIC-1 or PGE-M) assayable in relatively noninvasive specimens (e.g., blood, urine and stool) may be more facile to collect in clinical settings; however, such factors may lack relative specificity for neoplasia, In contrast, those biomarkers derived from tissue biopsies (e.g., *HPGD* expression) may offer greater specificity but require invasive procedures (e.g., endoscopic biopsy) for collection. Lastly, genetic markers, while likely to be more sensitive or specific depending on the strength of interaction with aspirin use and the allele frequencies within the population, may be more challenging to adopt as these tests may require additional interpretation and personalized genetic counseling. With the ASPIRED trial we aim to not only gain insight into the role of these markers in aspirin’s chemopreventive mechanism, but also to establish a measure of feasibility for assessing these markers clinically. Hopefully, the ASPIRED trial may serve as an early template for additional chemoprevention agent trials, especially as the field evolves to increasingly emphasize a molecular precision medicine approach for the prevention as well as treatment of disease.

### Trial status

Enrolling by invite only. To date (13 January 2017), 35 patients have completed both study visits, 13 patients have completed the baseline visit and are actively on treatment, five patients have been removed from the study for noncompliance (self-administered an off-study aspirin or NSAID) and one patient has been lost to follow-up. Twenty-six patients are scheduled for enrollment.

## Additional file

Additional file 1: SPIRIT checklist for ASPIRED trial. (PDF 508 kb)
Additional file 2: ASPIRED Consent Document. (PDF 80 kb)

## Abbreviations

ASPIRED: ASPirin Intervention for the REDuction of colorectal cancer risk
CRC: Colorectal cancer
DF/HCC: Dana-Farber/Harvard Cancer Center
MGH: Massachusetts General Hospital
NSAID: Nonsteroidal anti-inflammatory drug
OR: Odds ratio
PWS: Partial wave spectroscopic
RR: Relative risk

## References

[1] Siegel RL, Miller KD, Jemal A (2016). Cancer statistics, 2016. CA Cancer J Clin.

[2] Drew DA, Cao Y, Chan AT (2016). Aspirin and colorectal cancer: the promise of precision chemoprevention. Nat Rev Cancer.

[3] Bibbins-Domingo, K. on behalf of the United States Preventive Services Task Force. Aspirin Use for the Primary Prevention of Cardiovascular Disease and Colorectal Cancer: U.S. Preventive Services Task Force Recommendation Statement. Ann Intern Med. 2016;164(12):836–45. 10.7326/M16-0577 27064677

[4] Neale JR, Dean BJ (2008). Liquid chromatography-tandem mass spectrometric quantification of the dehydration product of tetranor PGE-M, the major urinary metabolite of prostaglandin E(2) in human urine. J Chromatogr B Analyt Technol Biomed Life Sci.

[5] Bertagnolli MM, Eagle CJ, Zauber AG, Redston M, Solomon SD, Kim K, Tang J, Rosenstein RB, Wittes J, Corle D (2006). Celecoxib for the prevention of sporadic colorectal adenomas. N Engl J Med.

[6] Benamouzig R, Deyra J, Martin A, Girard B, Jullian E, Piednoir B, Couturier D, Coste T, Little J, Chaussade S (2003). Daily soluble aspirin and prevention of colorectal adenoma recurrence: one-year results of the APACC trial. Gastroenterology.

[7] Bezawada NWK, Mehta RS, Song M, Milne GL, Ogino S, Fuchs C, Giovannucci E, Chan AT (2013). Urinary prostaglandin metabolites (PGE-M) are associated with risk of colorectal adenomas and chemopreventive response to anti-inflammatory drugs. Gastroenterology.

[8] Murphey LJ, Williams MK, Sanchez SC, Byrne LM, Csiki I, Oates JA, Johnson DH, Morrow JD (2004). Quantification of the major urinary metabolite of PGE2 by a liquid chromatographic/mass spectrometric assay: determination of cyclooxygenase-specific PGE2 synthesis in healthy humans and those with lung cancer. Anal Biochem.

[9] Chan AT, Ogino S, Fuchs CS (2007). Aspirin and the risk of colorectal cancer in relation to the expression of COX-2. N Engl J Med.

[10] Chan AT, Ogino S, Fuchs CS (2009). Aspirin use and survival after diagnosis of colorectal cancer. JAMA.

[11] Wang D, DuBois RN (2013). Urinary PGE-M: a promising cancer biomarker. Cancer Prev Res.

[12] Cai Q, Gao YT, Chow WH, Shu XO, Yang G, Ji BT, Wen W, Rothman N, Li HL, Morrow JD, Zheng W (2006). Prospective study of urinary prostaglandin E2 metabolite and colorectal cancer risk. J Clin Oncol.

[13] Shrubsole MJ, Cai Q, Wen W, Milne G, Smalley WE, Chen Z, Ness RM, Zheng W (2012). Urinary prostaglandin E2 metabolite and risk for colorectal adenoma. Cancer Prev Res.

[14] Johnson JC, Schmidt CR, Shrubsole MJ, Billheimer DD, Joshi PR, Morrow JD, Heslin MJ, Washington MK, Ness RM, Zheng W (2006). Urine PGE-M: a metabolite of prostaglandin E2 as a potential biomarker of advanced colorectal neoplasia. Clin Gastroenterol Hepatol.

[15] Morris PG, Zhou XK, Milne GL, Goldstein D, Hawks LC, Dang CT, Modi S, Fornier MN, Hudis CA, Dannenberg AJ (2013). Increased levels of urinary PGE-M, a biomarker of inflammation, occur in association with obesity, aging, and lung metastases in patients with breast cancer. Cancer Prev Res.

[16] Kim S, Taylor JA, Milne GL, Sandler DP (2013). Association between urinary prostaglandin E2 metabolite and breast cancer risk: a prospective, case-cohort study of postmenopausal women. Cancer Prev Res.

[17] Dong LM, Shu XO, Gao YT, Milne G, Ji BT, Yang G, Li HL, Rothman N, Zheng W, Chow WH, Abnet CC. Urinary prostaglandin E2 metabolite and gastric cancer risk in the Shanghai Women’s Health Study. Cancer Epidemiol Biomarkers Prev. 2009;18(11):3075–8. 10.1158/1055-9965.EPI-09-0680 PMC2783404 19861525

[18] Bottner M, Laaff M, Schechinger B, Rappold G, Unsicker K, Suter-Crazzolara C (1999). Characterization of the rat, mouse, and human genes of growth/differentiation factor-15/macrophage inhibiting cytokine-1 (GDF-15/MIC-1). Gene.

[19] Hromas R, Hufford M, Sutton J, Xu D, Li Y, Lu L (1997). PLAB, a novel placental bone morphogenetic protein. Biochim Biophys Acta.

[20] Paralkar VM, Vail AL, Grasser WA, Brown TA, Xu H, Vukicevic S, Ke HZ, Qi H, Owen TA, Thompson DD (1998). Cloning and characterization of a novel member of the transforming growth factor-beta/bone morphogenetic protein family. J Biol Chem.

[21] Wang X, Baek SJ, Eling TE (2013). The diverse roles of nonsteroidal anti-inflammatory drug activated gene (NAG-1/GDF15) in cancer. Biochem Pharmacol.

[22] Breit SN, Johnen H, Cook AD, Tsai VW, Mohammad MG, Kuffner T, Zhang HP, Marquis CP, Jiang L, Lockwood G (2011). The TGF-beta superfamily cytokine, MIC-1/GDF15: a pleotrophic cytokine with roles in inflammation, cancer and metabolism. Growth Factors.

[23] Brown DA, Ward RL, Buckhaults P, Liu T, Romans KE, Hawkins NJ, Bauskin AR, Kinzler KW, Vogelstein B, Breit SN (2003). MIC-1 serum level and genotype: associations with progress and prognosis of colorectal carcinoma. Clin Cancer Res.

[24] Bauskin AR, Brown DA, Kuffner T, Johnen H, Luo XW, Hunter M, Breit SN (2006). Role of macrophage inhibitory cytokine-1 in tumorigenesis and diagnosis of cancer. Cancer Res.

[25] Brown J, Delaine C, Zaccheo OJ, Siebold C, Gilbert RJ, van Boxel G, Denley A, Wallace JC, Hassan AB, Forbes BE, Jones EY (2008). Structure and functional analysis of the IGF-II/IGF2R interaction. EMBO J.

[26] Zimmers TA, Gutierrez JC, Koniaris LG (2010). Loss of GDF-15 abolishes sulindac chemoprevention in the ApcMin/+ mouse model of intestinal cancer. J Cancer Res Clin Oncol.

[27] Wang X, Kingsley PJ, Marnett LJ, Eling TE (2011). The role of NAG-1/GDF15 in the inhibition of intestinal polyps in APC/Min mice by sulindac. Cancer Prev Res.

[28] Baek SJ, Okazaki R, Lee SH, Martinez J, Kim JS, Yamaguchi K, Mishina Y, Martin DW, Shoieb A, McEntee MF, Eling TE (2006). Nonsteroidal anti-inflammatory drug-activated gene-1 over expression in transgenic mice suppresses intestinal neoplasia. Gastroenterology.

[29] Mehta RS, Song M, Bezawada N, Wu K, Garcia-Albeniz X, Morikawa T, Fuchs CS, Ogino S, Giovannucci EL, Chan AT. A prospective study of macrophage inhibitory cytokine-1 (MIC-1/GDF15) and risk of colorectal cancer. J Natl Cancer Inst. 2014;106(4). doi:10.1093/jnci/dju016. 10.1093/jnci/dju016 PMC3982884 24565956

[30] Polakis P (1999). The oncogenic activation of beta-catenin. Curr Opin Genet Dev.

[31] Waltzer L, Bienz M (1999). The control of beta-catenin and TCF during embryonic development and cancer. Cancer Metastasis Rev.

[32] Behrens J (2000). Control of beta-catenin signaling in tumor development. Ann N Y Acad Sci.

[33] Bos CL, Kodach LL, van den Brink GR, Diks SH, van Santen MM, Richel DJ, Peppelenbosch MP, Hardwick JC (2006). Effect of aspirin on the Wnt/beta-catenin pathway is mediated via protein phosphatase 2A. Oncogene.

[34] Dihlmann S, Siermann A, von Knebel DM (2001). The nonsteroidal anti-inflammatory drugs aspirin and indomethacin attenuate beta-catenin/TCF-4 signaling. Oncogene.

[35] Greenspan EJ, Madigan JP, Boardman LA, Rosenberg DW (2011). Ibuprofen inhibits activation of nuclear {beta}-catenin in human colon adenomas and induces the phosphorylation of GSK-3{beta}. Cancer Prev Res (Phila).

[36] Dihlmann S, Klein S, Doeberitz MM (2003). Reduction of beta-catenin/T-cell transcription factor signaling by aspirin and indomethacin is caused by an increased stabilization of phosphorylated beta-catenin. Mol Cancer Ther.

[37] Goessling W, North TE, Loewer S, Lord AM, Lee S, Stoick-Cooper CL, Weidinger G, Puder M, Daley GQ, Moon RT, Zon LI (2009). Genetic interaction of PGE2 and Wnt signaling regulates developmental specification of stem cells and regeneration. Cell.

[38] Castellone MD, Teramoto H, Gutkind JS (2006). Cyclooxygenase-2 and colorectal cancer chemoprevention: the beta-catenin connection. Cancer Res.

[39] Castellone MD, Teramoto H, Williams BO, Druey KM, Gutkind JS (2005). Prostaglandin E2 promotes colon cancer cell growth through a Gs-axin-beta-catenin signaling axis. Science.

[40] Buchanan FG, DuBois RN (2006). Connecting COX-2 and Wnt in cancer. Cancer Cell.

[41] Clevers H (2006). Colon cancer—understanding how NSAIDs work. N Engl J Med.

[42] Shao J, Jung C, Liu C, Sheng H (2005). Prostaglandin E2 Stimulates the beta-catenin/T cell factor-dependent transcription in colon cancer. J Biol Chem.

[43] Nan H, Morikawa T, Suuriniemi M, Imamura Y, Werner L, Kuchiba A, Yamauchi M, Hunter DJ, Kraft P, Giovannucci EL (2013). Aspirin use, 8q24 single nucleotide polymorphism rs6983267, and colorectal cancer according to CTNNB1 alterations. J Natl Cancer Inst.

[44] Tenesa A, Farrington SM, Prendergast JG, Porteous ME, Walker M, Haq N, Barnetson RA, Theodoratou E, Cetnarskyj R, Cartwright N (2008). Genome-wide association scan identifies a colorectal cancer susceptibility locus on 11q23 and replicates risk loci at 8q24 and 18q21. Nat Genet.

[45] Tomlinson I, Webb E, Carvajal-Carmona L, Broderick P, Kemp Z, Spain S, Penegar S, Chandler I, Gorman M, Wood W (2007). A genome-wide association scan of tag SNPs identifies a susceptibility variant for colorectal cancer at 8q24.21.. Nat Genet.

[46] Zanke BW, Greenwood CM, Rangrej J, Kustra R, Tenesa A, Farrington SM, Prendergast J, Olschwang S, Chiang T, Crowdy E (2007). Genome-wide association scan identifies a colorectal cancer susceptibility locus on chromosome 8q24. Nat Genet.

[47] Pomerantz MM, Ahmadiyeh N, Jia L, Herman P, Verzi MP, Doddapaneni H, Beckwith CA, Chan JA, Hills A, Davis M (2009). The 8q24 cancer risk variant rs6983267 shows long-range interaction with MYC in colorectal cancer. Nat Genet.

[48] Sur IK, Hallikas O, Vaharautio A, Yan J, Turunen M, Enge M, Taipale M, Karhu A, Aaltonen LA, Taipale J (2012). Mice lacking a Myc enhancer that includes human SNP rs6983267 are resistant to intestinal tumors. Science.

[49] Ensor CM, Tai HH (1995). 15-hydroxyprostaglandin dehydrogenase. J Lipid Mediat Cell Signal.

[50] Liu T, Ortiz JA, Taing L, Meyer CA, Lee B, Zhang Y, Shin H, Wong SS, Ma J, Lei Y (2011). Cistrome: an integrative platform for transcriptional regulation studies. Genome Biol.

[51] Backlund MG, Mann JR, Holla VR, Buchanan FG, Tai HH, Musiek ES, Milne GL, Katkuri S, DuBois RN (2005). 15-hydroxyprostaglandin dehydrogenase is down-regulated in colorectal cancer. J Biol Chem.

[52] Yan M, Rerko RM, Platzer P, Dawson D, Willis J, Tong M, Lawrence E, Lutterbaugh J, Lu S, Willson JK (2004). 15-hydroxyprostaglandin dehydrogenase, a COX-2 oncogene antagonist, is a TGF-beta-induced suppressor of human gastrointestinal cancers. Proc Natl Acad Sci U S A.

[53] Myung SJ, Rerko RM, Yan M, Platzer P, Guda K, Dotson A, Lawrence E, Dannenberg AJ, Lovgren AK, Luo G (2006). 15-hydroxyprostaglandin dehydrogenase is an in vivo suppressor of colon tumorigenesis. Proc Natl Acad Sci U S A.

[54] Yan M, Myung SJ, Fink SP, Lawrence E, Lutterbaugh J, Yang P, Zhou X, Liu D, Rerko RM, Willis J (2009). 15-hydroxyprostaglandin dehydrogenase inactivation as a mechanism of resistance to celecoxib chemoprevention of colon tumors. Proc Natl Acad Sci U S A.

[55] Thompson CL, Fink SP, Lutterbaugh JD, Elston RC, Veigl ML, Markowitz SD, Li L (2013). Genetic variation in 15-hydroxyprostaglandin dehydrogenase and colon cancer susceptibility. PLoS One.

[56] Roberts HR, Smartt HJ, Greenhough A, Moore AE, Williams AC, Paraskeva C (2011). Colon tumour cells increase PGE(2) by regulating COX-2 and 15-PGDH to promote survival during the microenvironmental stress of glucose deprivation. Carcinogenesis.

[57] Fink S YM, Nishihara R, Jung S, Kuchiba A, Wu K, Cho E, Giovannucci E, Fuchs C, Ogino S, Markowitz SD, Chan AT. Aspirin and the risk of colorectal cancer in relation to expression of 15-hydroxyprostaglandin dehydrogenase expression. Sci Transl Med 2014; In Press. 10.1126/scitranslmed.3008481 PMC4030641 24760190

[58] Fink SP, Yang DH, Barnholtz-Sloan JS, Ryu YM, Mikkola D, Potter JD, Lampe JW, Markowitz SD, Myung SJ (2013). Colonic 15-PGDH levels are stable across distance and time and are not perturbed by aspirin intervention. Dig Dis Sci.

[59] Smartt HJ, Greenhough A, Ordonez-Moran P, Talero E, Cherry CA, Wallam CA, Parry L, Al Kharusi M, Roberts HR, Mariadason JM (2012). beta-catenin represses expression of the tumour suppressor 15-prostaglandin dehydrogenase in the normal intestinal epithelium and colorectal tumour cells. Gut.

[60] Ellis RJ, Minton AP (2003). Join the crowd. Nature.

[61] Boyle JO, Gumus ZH, Kacker A, Choksi VL, Bocker JM, Zhou XK, Yantiss RK, Hughes DB, Du B, Judson BL (2010). Effects of cigarette smoke on the human oral mucosal transcriptome. Cancer Prev Res (Phila).

[62] Misteli T, Soutoglou E (2009). The emerging role of nuclear architecture in DNA repair and genome maintenance. Nat Rev.

[63] Subramanian H, Pradhan P, Liu Y, Capoglu IR, Li X, Rogers JD, Heifetz A, Kunte D, Roy HK, Taflove A, Backman V (2008). Optical methodology for detecting histologically unapparent nanoscale consequences of genetic alterations in biological cells. PNAS.

[64] Roy HK, Turzhitsky V, Wali R, Radosevich AJ, Jovanovic B, Della’Zanna G, Umar A, Rubin DT, Goldberg MJ, Bianchi L, et al. Spectral biomarkers for chemoprevention of colonic neoplasia: a placebo-controlled double-blinded trial with aspirin. Gut. 2017;66(2):285–292. 10.1136/gutjnl-2015-309996 PMC5108693 26503631

[65] Nelson AM, Walk ST, Taube S, Taniuchi M, Houpt ER, Wobus CE, Young VB (2012). Disruption of the human gut microbiota following Norovirus infection. PLoS One.

[66] Vujkovic-Cvijin I, Dunham RM, Iwai S, Maher MC, Albright RG, Broadhurst MJ, Hernandez RD, Lederman MM, Huang Y, Somsouk M (2013). Dysbiosis of the gut microbiota is associated with HIV disease progression and tryptophan catabolism. Sci Transl Med.

[67] Ahn J, Sinha R, Pei Z, Dominianni C, Wu J, Shi J, Goedert JJ, Hayes RB, Yang L (2013). Human gut microbiome and risk for colorectal cancer. J Natl Cancer Inst.

[68] Kostic AD, Gevers D, Pedamallu CS, Michaud M, Duke F, Earl AM, Ojesina AI, Jung J, Bass AJ, Tabernero J (2012). Genomic analysis identifies association of *Fusobacterium* with colorectal carcinoma. Genome Res.

[69] Zhu Q, Gao R, Wu W, Qin H (2013). The role of gut microbiota in the pathogenesis of colorectal cancer. Tumour Biol.

[70] Biarc J, Nguyen IS, Pini A, Gosse F, Richert S, Thierse D, Van Dorsselaer A, Leize-Wagner E, Raul F, Klein JP, Scholler-Guinard M (2004). Carcinogenic properties of proteins with pro-inflammatory activity from *Streptococcus infantarius* (formerly *S. bovis*). Carcinogenesis.

[71] Ellmerich S, Scholler M, Duranton B, Gosse F, Galluser M, Klein JP, Raul F (2000). Promotion of intestinal carcinogenesis by *Streptococcus bovis*. Carcinogenesis.

[72] Wang X, Huycke MM (2007). Extracellular superoxide production by *Enterococcus faecalis* promotes chromosomal instability in mammalian cells. Gastroenterology.

