# Supplementary material for: ASPirin Intervention for the REDuction of colorectal cancer risk (ASPIRED): a study protocol for a randomized controlled trial
Source: Trials. 2017 Feb 1;18:50. doi: 10.1186/s13063-016-1744-z (PMC5286828; doi:10.1186/s13063-016-1744-z)
Supplement: Additional file 2: — ASPIRED Consent Document. (PDF 80 kb) [file 13063_2016_1744_MOESM2_ESM.pdf]

Research Consent Form
for Biomedical Research
Dana-Farber/ Harvard Cancer Center
BIDMC/BCH/BWH/DFCI/MGH/Partners Network Affiliates OHRS 04.07.14

Protocol Title: ASPIRED: ASPirin Intervention for the REDuction of Colorectal Cancer Risk

National Cancer Institute Protocol #:

DF/HCC Principal Research Doctor / Institution: Andrew T. Chan, MD, MPH / Massachusetts General Hospital

DF/HCC Site-Responsible Research Doctor(s) / Institution(s):
Donna Spiegelman, ScD / Harvard School of Public Health

Matthew Freedman, MD / Dana Farber Cancer Institute, Broad Institute

A. INTRODUCTION

You are invited to take part in a clinical trial, a type of research study, because you have previously undergone a colonoscopy at Massachusetts General Hospital and had an adenoma removed during this previous procedure. This research study is studying a drug intervention as a possible chemoprevention strategy for colorectal cancer.

The names of the study interventions involved in this study are:
- A daily aspirin regimen.

For purposes of this research, you will be referred to as a "participant".

It is expected that about 180 people will take part in this research study.

The National Cancer Institute (NCI) of the National Institutes of Health (NIH) is supporting this research study by providing funding for the research study.

This research consent form explains why this research study is being done, what is involved in participating in the research study, the possible risks and benefits of participation, alternatives to participation, and your rights as a research participant. The decision to participate is yours. If you decide to participate, please sign and date at the end of this form. We will give you a copy so that you

can refer to it while you are involved in this research study. If you choose not to participate in this research study, the research doctors will discuss other treatment options with you and/or refer you back to your primary doctor.

We encourage you to take some time to think this over, to discuss it with other people and your primary doctor, and to ask questions now and at any time in the future.

B. WHY IS THIS RESEARCH STUDY BEING DONE?

This research study is a chemoprevention clinical trial, designed to test the safety and effectiveness of an investigational intervention to learn whether the intervention works in treating a specific disease. “Investigational” means that the intervention is being studied.

In this research study, we are investigating the use of aspirin as a potential chemopreventive agent to reduce risk of colorectal cancer. Aspirin is part of the non-steroidal anti-inflammatory drug (NSAID) family, which are drugs routinely used for their pain-killing (analgesic), fever-reducing (antipyretic), or anti-inflammatory properties. Most NSAIDs are available as over-the-counter formulations. Substantial evidence has conclusively demonstrated that aspirin reduces the risk of colorectal neoplasia, yet there remains uncertainty surrounding its mode of action. Aspirin has already been established to reduce the risk of cardiovascular disease. Prospective studies as well as randomized clinical trials demonstrate that aspirin reduces the risk of precancerous polyps and colorectal cancer.

As previously stated, the exact mechanism by which aspirin acts to prevent colorectal cancer is still unknown. However, it is believed that aspirin may prevent colorectal cancer through multiple interrelated biological mechanisms including the reduction of chronic inflammation, a known risk factor for colorectal cancer. Aspirin has been shown to directly effect prostaglandins, a class of biologic molecules that play important roles in controlling the normal inflammatory responses within your body. By performing this research study, we hope to define the optimal dose of aspirin administration and study the mechanisms of its anti-cancer effect, which may lead to the discovery of novel specific characteristics (markers) that can be used to select patients for aspirin treatment.

C. WHAT OTHER OPTIONS ARE THERE?

Taking part in this research study is voluntary. Instead of being in this research study, you have other options which may include the following:

- Receive standard treatment including follow-up colonoscopy at the appropriate surveillance interval as prescribed by your physicians
- Take part in another research study.
- Receive the same drugs, but not as part of a research study.

Please talk to the research doctor about your options before you decide whether you will take part in this research study.

D. WHAT IS INVOLVED IN THE RESEARCH STUDY?

Because no one knows which of the study options is best, you will be "randomized" into one of the study groups: placebo (no aspirin), low dose aspirin (81 mg/day), or standard dose aspirin (325 mg/day). Randomization means that you are put into a group by chance. It is like flipping a coin. Neither you nor the research doctor will choose what group you will be in. Neither you nor the research doctor will know what group you are in. You will have a one in three chance of being placed in any of the following groups:

- Arm A: Daily Placebo (no aspirin) for the duration of the study.
- Arm B: Daily low dose aspirin (81 mg/day) for the duration of the study.
- Arm C: Daily standard dose aspirin (325 mg/day) for the duration of the study.

You will be given a study medication and it will contain either aspirin or placebo (pills with no medicine).

Sometimes it is hard to keep track of all of the details and procedures that are part of a research study. We will describe them in this consent form and you can refer to this at any time during the research study.

A study coordinator will contact you, by phone, weekly to ensure that you taking the drug as directed.

Before the research starts (screening):

If you choose to take part in this study, we will ask you to sign this consent form when you come in for your first study visit. You will be contacted by or you will have contacted a study coordinator prior to your initial visit to preliminarily determine your eligibility based on information you provide voluntarily or is included in your longitudinal medical record.

After signing this consent form, you may be asked to undergo some screening tests or procedures to find out if you can be in the research study. Many of these tests and procedures are likely to be part of regular care. If you have had some of these tests or procedures recently, they may or may not have to be repeated.

- **A medical history,** which includes questions about your health, current medications, and any allergies.
- **Performance status**, which evaluates how you are able to carry on with your usual activities.

If these tests confirm that you are eligible to participate in the research study, you will begin the study treatment. If you do not meet the eligibility criteria, you will not be able to participate in this research study.

Study Visit 1: Initial (baseline) Visit

At the baseline visit, your physician will obtain written, informed consent for the study as well as a standard clinical consent to perform a flexible sigmoidoscopy. This flexible sigmoidoscopy is similar to your previous colonoscopy. During the procedure the endoscope is only inserted a few inches past your rectum. This flexible sigmoidoscopy will be performed without a bowel preparation, which means that you do not have to drink any laxative preparation in advance of the procedure. At this visit, you will undergo measurements of height, weight, waist and hip circumference and provide a blood, saliva, and urine specimen. A study gastroenterologist will then peform the flexible sigmoidoscopy, advancing only about 6-10 inches. Thus, you will not be requiring any sedation for this procedure. No more than a total of 24 mucosal biopsies will be taken from the rectum and sigmoid colon and immediately placed in collection tubes.

This visit will involve the following:

- **Oral Study Drug(s):** The treatment lasts at minimum 8 weeks during which time you will be taking the study drug 1 time per day with food and a full glass of water. This may continue up to 12 weeks.
- **Clinical exam:** During the baseline visit you will have a physical exam and you will be asked questions about your general health and specific questions about any problems that you might be having and any medications you may be taking.
- **A lifestyle and food frequency questionnaire.** Our research assistant will help you complete a questionnaire, which evaluates your lifestyle risk factors, current nutritional intake, and dietary trends.
- **Blood samples drawn.** Approximately 2 tablespoons of blood (30 mLs)
- **Saliva sample provided.**
- **Urine sample provided**.
- **Flexible sigmoidoscopy,** where **up to 24 normal colon tissue biopsies** will be collected.
- **Endoscopic cytology brushing**, where we will insert a small brush through the endoscope during sigmoidoscopy to collect some cells.
- **Stool specimen collected** (during flexible sigmoidoscopy)
- **Confirm or schedule final visit,** which will occur at least 8 weeks but no more than 12 weeks following this initial visit.

<u>Study Visit 2</u>: Final Visit, study treatment ends

This visit will involve the following:

- **Return drug bottle with any remaining pills.**
- **Clinical exams*:*** During this visit you will have a brief physical exam and you will be asked questions about your general health and specific questions about any problems that you might be having and any medications you may be taking.
- **A second lifestyle and food frequency questionnaire**
- **Blood samples drawn.** Approximately 2 tablespoons of blood (30 mLs)
- **Saliva sample provided.**
- **Urine sample provided**.
- **Flexible sigmoidoscopy,** where **up to 24 normal colon tissue biopsies** will be collected.
- **Endoscopic cytology brushing**
- **Stool specimen collected** (during flexible sigmoidoscopy)

Research Study Plan:

	Visit 1<br>Initial Visit	Visit 2<br>Final Visit
Medical History & Physical Exam	X	X
Blood Draw	X	X
Saliva Collection	X	X
Urine Collection	X	X
Flexible Sigmoidoscopy	X	X
Normal Colon Tissue Biopsy	X	X
Cytological Brushing	X	X
Complete Questionnaires	X	X
Stool collection	X	X
Pregnancy Test	X	X
Receive study drug[a]	X	
Return pill bottle with unused drug capsules		X
a: You will take unmarked capsules of study drug (or placebo) once daily until your final visit 8-12 weeks after your initial visit.		

Planned Follow-up:

Between the initial and final visits a study coordinator will contact you weekly to make sure you aren't experiencing any adverse side effects and track your use of the study drug. After the study we may also contact you periodically (no more than 1-2 times annually) to follow-up on additional information including any continued aspirin use and results of any follow-up colonoscopies. After the study is completed, we may contact you with the contact information you provided for future related studies.

Use and Storage of Your Samples:

We will use your urine, blood, stool, saliva, and tissue samples to measure the effect of aspirin on certain biological compounds found in these specimens. Those biological mechanisms impacted include but are not limited to prostaglandins (measured in urine) and inflammatory markers (measured in blood serum), important molecules that may provide an indication as to whether a person would benefit from using aspirin to prevent colorectal cancer.

We will perform genetic research on the DNA and RNA in your tissue and stool samples. DNA (deoxyribonucleic acid) is the material that makes up your genes. All living things are made of cells. Genes are the part of cells that contain the instructions, which tell our bodies how to grow and work, and determine physical characteristics like hair and eye color. Changes or differences in a gene may affect a person's chances of developing a particular disease, or how a person might respond to a particular drug, like aspirin. RNA (ribonucleic acid) is a molecule that carries instructions from DNA about how to make proteins to the parts of the cell that actually make the proteins. Differences in cellular RNA can also provide insight into an individual's risk for a disease or potential for response to a drug. We will do these studies using your tissue biopsies. From your stool, we will analyze the DNA, RNA, and proteins that are specific to the bacterial populations that live within your gut. By doing so, we can determine if the genes of these bacteria affect your health or are affected by aspirin treatment.

We may also access archived tissue specimens such as polyps or adenomas that were removed during your most recent colonoscopy prior to the start of this study to correlate our findings with tissue-specific markers of colorectal cancer. Periodically, we may examine your longitudinal medical record to check for a diagnosis of any new digestive diseases or alterations in aspirin use.

In order to allow researchers to share test results, the National Institutes of Health (NIH) and other central repositories have developed special data (information) banks that analyze data and collect the results of genetic studies. We do not think that there will be further risks to your privacy and confidentiality by sharing your genetic information with these banks. However, we cannot predict how genetic information will be used in the future. The data will be de-identified. Your name or other directly identifiable information will never be given to central banks. There are many safeguards in place to protect your information while they are stored in repositories and used for research.

Blood, stool, urine, saliva, and gastrointestinal tract tissue samples may be frozen and stored indefinitely for future research at the MGH/DFHCC. All specimens will be coded with a number and date, and your name will never be used. The investigators will keep the link between your identity and the code number in a secure location and access will be limited to only essential study staff. Your samples may also be used in future research projects. It is not possible to list every research project. Also, we cannot predict all of the research questions that will be important over the next years. As we learn more, new types of research and new research questions may arise. Any information you provide and the results of your analyses are considered strictly confidential and are used for medical statistical purposes only. Results from future research using these samples may appear in publications and at meetings but your name or other identifying information will not.

MGH will maintain these coded samples indefinitely or until the samples are exhausted. MGH will assert all rights of ownership in the samples. Research done with the samples may be used to develop new products in the future. In this event, the MGH and/or the developers will assert all rights arising from use of the samples.

Please know that if the investigator leaves the institution, the research and the tissue might remain at the DF/HCC or might be transferred to another institution.

Specimens Sent to Outside Collaborators

Coded samples and/or data may be sent by MGH/DFHCC to other researchers who are also studying aspirin chemoprevention and/or collaborating with the DFHCC/MGH including but not limited to the National Institutes of Health, the Ragon Institute, the Dana Farber Cancer Institute, Children's Hospital, Vanderbilt University, the Broad Institute and the Harvard School of Public Health. All other scientists and/or collaborators must meet MGH requirements for sharing samples and/or data including treating the data or materials as medically confidential, obtaining approval from their Human Subjects review boards, and agreeing not to share the data or materials with other parties. Affiliated researchers or laboratories outside of MGH/DFHCC will never know who you are nor have access to the code linking the samples to you.

E. HOW LONG WILL I BE IN THIS RESEARCH STUDY?

You will be in this research study for a minimum of 8 weeks and maximum of 12 weeks.

The research doctor may decide to take you off the research study for many reasons including if:

- It is considered to be in your best interest
- The study treatment or procedures are found to be unsafe or ineffective
- There is any problem with following study treatments and procedures
- Your condition worsens
- A decision is made to close the study
- Or for other unforeseen reasons that make it necessary to stop your participation in the research study
- You use non-study aspirin or other NSAID.

If you are removed from the research study, the research doctor will explain to you why you were removed.

In addition, you can stop participating in the research study at any time, however, the FDA requires that any information collected up to the point of your withdrawal cannot be removed from the study. If you decide to stop participating in this research study, we encourage you to talk to the research doctor and your primary doctor first.

F. WHAT ARE THE RISKS OR DISCOMFORTS OF THE RESEARCH STUDY?

There are risks to taking part in any research study. One risk is that you may get a dose of study drug that does not provide you any preventative benefit. Another risk is that there may be side effects.

All treatments can have side effects, which can range from mild and reversible to severe, long lasting and possibly life-threatening. There is a great deal of variability among side effects of different treatments and between individuals. In a research study, all of the risks or side effects may not be known before you start the study. **You need to tell your doctor or a member of the study team immediately if you experience any side effects.**

Everyone in the research study will be watched carefully for side effects. If you experience side effects, they may go away after you stop taking the study drug. Some side effects can be mild; but others can be long lasting and may never go away. Some may be life-threatening or fatal.

Since the effect of the study drug(s) taken with other medications may not be known, it is important that you tell the research doctor about all prescription and non-prescription drugs, herbal preparations and nutritional supplements that you are taking or planning to take. There may also be some foods that you should avoid while on this research study and your research doctor will review this information with you.

During the research study, you will be notified of newly discovered side effects or significant findings, which may affect your health or willingness to participate. You may be asked to sign a new consent form that shows that you have been informed of new information relating to this research study.

Risks Associated with Aspirin intervention:

Rare

Less than 1% of people may develop serious side effects from aspirin use including:

- Bleeding
- Formation of ulcers
- Kidney damage
- Allergic reaction to aspirin
- Very rarely, cause the type of stroke that results from bleeding inside the head.

The following describes the possible side effects of procedures done only for the purposes of research.

Risks Associated with Flexible Sigmoidoscopy:
Flexible sigmoidoscopy is generally a safe test although rare complications can occur. These can include some mild pain or discomfort, like a feeling of fullness, felt during this test. Even more rarely (less than 1 in 10,000 times), a hole (perforation) can be made in the side of the rectum or colon that can require surgical intervention.

Risks Associated with Biopsies:
Biopsies of the colon are performed by inserting biopsy forceps through the endoscope and removing a small piece of tissue. Minor bleeding or pain, the most common complications resulting from endoscopic biopsy, complicate less than 1 of every 3000 (0.03%) colonoscopic procedures. The risks may include:

- Pain and discomfort. The amount of pain and discomfort will vary, but should be minimal. These risks can be discussed with the study doctor.
- Minor bleeding at the biopsy site. Major bleeding episodes that require blood transfusions or hospitalization are extremely rare.
- This study requires 24 biopsies per procedure. A study of the safety of research biopsies by the National Institutes of Health has determined that more than 20 biopsies per procedure, including as many as 85, is well tolerated and appears to have no more than minimal risk without increasing the risk of otherwise routine colonoscopy. Furthermore, the same study found no specific increase in risk of complications due to the use of NSAIDs, including aspirin.Tenderness at the biopsy site.
- Scarring at the biopsy site.
- Rarely, an infection at the biopsy site.

Rarely, complications from intestinal biopsies can be life threatening. As with any interventional procedure, other potentially serious complications from bleeding or organ damage may occur. These might require additional surgical intervention.

Risks Associated with Phlebotomy:
You will have 2 blood draws during the study. The risk of blood draws include discomfort at the site of blood draw, bruising, bleeding, infection, and rarely fainting. A small amount of blood (30 mLs or approximately 2 tablespoons) will be taken at each blood draw and poses minimal risk.

Reproductive Risks:
The drugs used in this research study may affect a fetus. While participating in this research study, you should not become pregnant or father a baby, and should not nurse a baby. Let your doctor know immediately if you become pregnant or find out that you are going to be the father of a child. We can provide counseling about preventing pregnancy for either male or female study participants.

In the event that your partner becomes pregnant, it may be critical to share information regarding your participation in this research study with that person. Your research doctor should also be told if this happens. The study sponsor may want to collect data on your partner's pregnancy.

Risks Associated with Genetic Research

Genetic research studies may create special risks to human subjects and their relatives. These involve medical, psychosocial and economic risks, such as the possible loss of privacy, insurability and employability, change in immigration status and limits on education options, and may create a social stigma. Knowledge of one's genetic make-up may also affect one's knowledge of the disease risk status of family members. To minimize the risks associated with genetic testing, no results will be filed in the subject's medical record and no research results will be given to subjects or healthcare providers. Any genetic information entered into research databases will always be coded and will not be identified with your name or other identifiable personal health information.

Non-Physical Risks:

Because of side effects or the time required for tests and clinic visits while you are on this research study, you may be unable to keep up with your normal daily activities. Each study visit should last no more than 4 hours each.

The questionnaires used in this study may be upsetting. If you find the questionnaires upsetting, you may speak with the research doctor or ask to be referred for additional emotional support.

G. WHAT ARE THE BENEFITS OF THE RESEARCH STUDY?

You may or may not benefit from this research study. If aspirin is effective in preventing colorectal cancer; you may receive some benefit while you are taking the medication. It is not known whether any possible benefit would persist when the treatment is stopped. We will not be able to tell from this study whether your personal risk of developing colorectal cancer has been lowered. This study may help researchers develop a new way to determine which people may benefit from aspirin therapy for the prevention of colorectal cancer.

H. CAN I STOP BEING IN THE RESEARCH STUDY AND WHAT ARE MY RIGHTS?

You have the right to choose not to sign this form. If you decide not to sign this form, you cannot participate in this research study.

You can stop being in the research study at any time. Tell the research doctor if you are thinking about stopping or decide to stop. He or she will tell you how to stop. Leaving the research study will not affect your medical care outside of the research study.

If you choose not to participate, are not eligible to participate, or withdraw from this research study, this will not affect your present or future care and will not cause any penalty or loss of benefits to which you are otherwise entitled.

It is important to tell the research doctor if you are thinking about stopping so your research doctor can evaluate the risks from stopping the drug intervention. Another reason to tell your research doctor that you are thinking about stopping is to discuss what follow-up care and testing could be most helpful for you.

I. WILL I BE PAID TO TAKE PART IN THIS RESEARCH STUDY?

You will be compensated for participating in this research study. Participants who complete the baseline visit and protocol will receive $200.00 (US) compensation. Patients who complete the end visit and protocol will receive an additional $300.00 (US) compensation. Additionally, if you require parking for your two study visits, you will receive up to four hours of parking at no charge to you.

We may use your samples and information to develop a new product or medical test to be sold. The sponsor and hospital may benefit if this happens. There are no plans to pay you if your samples are used for this purpose.

J. WHAT ARE THE COSTS?

There are no out-of-pocket expenses that will be charged to you relating to this study. You will not be charged for aspirin or for the study procedures.

If you have questions about your insurance coverage, or the items you might be required to pay for, please call financial services for information. The contact information for financial services are:

- Massachusetts General Hospital: (617) 726-2191

The National Cancer Institute provides an online resource to help people participating in cancer clinical trials understand which services their insurance company is required by law to pay. This can be found at the website below or can be provided by the study team:

www.cancer.gov or 1-800-4-CANCER (1-800-422-6237)

K. WHAT HAPPENS IF I AM INJURED OR SICK BECAUSE I TOOK PART IN THIS RESEARCH STUDY?

If you think you have been injured as a result of taking part in this research study, tell the person in charge of this research study as soon as possible. The research doctor's name and phone number are listed in this consent form.

We will offer you the care needed to treat injuries directly resulting from taking part in this research. We may bill your insurance company or other third parties, if appropriate, for the costs of the care you get for the injury, but you may also be responsible for some of them.

There are no plans for any of the sponsors of this study to pay you or give you other compensation for the injury. You do not give up your legal rights by signing this form.

L. WHAT ABOUT CONFIDENTIALITY?

We will take measures to protect the privacy and security of all your personal information, but we cannot guarantee complete confidentiality of study data.

Medical information created by this research study may become part of a DF/HCC research database.

The results of this research study may be published. You will not be identified in publications without your permission.

A description of this clinical trial will be available on http://www.ClinicalTrials.gov, as required by U.S. Law. This Web site will not include information that can identify you. At most, the Web site will include a summary of the results. You can search this Web site at any time.

M. WHOM DO I CONTACT IF I HAVE QUESTIONS ABOUT THE RESEARCH STUDY?

If you have questions about the study, please contact the research doctor or study staff as listed below:

Massachusetts General Hospital

- Andrew T. Chan, MD, MPH: (617)726-3212
- Dan Chung, MD: (617)726-3544
- Manish Gala, MD: (617)506-9644
- David A. Drew, Ph.D.: (617)724-7360
- Katherine Gilpin: (617)724-1326

If you need to contact study staff outside normal business hours, please contact:
24-Hour Contact: Andrew Chan, MD at (617)726-7777, pager 31100.

For questions about your rights as a research participant, please contact a representative of the Office for Human Research Studies at DFCI (617) 632-3029. This can include questions about your participation in the study, concerns about the study, a research related injury, or if you feel/felt under pressure to enroll in this research study or to continue to participate in this research study.

N. PRIVACY OF PROTECTED HEALTH INFORMATION

Federal law requires Dana-Farber/Harvard Cancer Center (DF/HCC) and its affiliated research doctors, health care providers, and physician network to protect the privacy of information that identifies you and relates to your past, present, and future physical and mental health conditions ("protected health information"). If you enroll in this research study, your "protected health information" will be used and shared with others as explained below.

1. What protected health information about me will be used or shared with others during this research?

- Existing medical records, including mental health records.

- New health information created from study-related tests, procedures, visits, and/or questionnaires

2. Why will protected information about me be used or shared with others?

The main reasons include the following:

- To conduct and oversee the research described earlier in this form;
- To ensure the research meets legal, institutional, and accreditation requirements;
- To conduct public health activities (including reporting of adverse events or situations where you or others may be at risk of harm); and
- To provide the study sponsor with information arising from an adverse event or other event that relates to the safety or toxicity of the drug for the purpose of this or other research relating the study drug and its use in cancer; and,
- Other reasons may include for treatment, payment, or health care operations. For example, some medical information produced by this research study may become part of your hospital medical record because the information may be necessary for your medical care. (You will also be given a notice for use and sharing of protected health information.)

3. Who will use or share protected health information about me?

- DF/HCC and its affiliated research doctors and entities participating in the research will use and share your protected health information. In addition, other DF/HCC offices that deal with research oversight, billing or quality assurance will be able to use and share your protected health information.

4. With whom outside of DF/HCC may my protected health information be shared?

While all reasonable efforts will be made to protect the confidentiality of your protected health information, it may also be shared with the following entities:

- Outside individuals or entities that have a need to access this information to perform functions relating to the conduct of this research such as analysis by outside laboratories on behalf of DF/HCC and its affiliates (for example, data storage companies, insurers, or legal advisors).

- The sponsor(s) of the study, its subcontractors, and its agent(s): National Cancer Institute and National Institutes of Health
- Other research doctors and medical centers participating in this research.
- Federal and state agencies (for example, the Department of Health and Human Services, the Food and Drug Administration, the National Institutes of Health, and/or the Office for Human Research Protections), or other domestic or foreign government bodies if required by law and/or necessary for oversight purposes. A qualified representative of the FDA and the National Cancer Institute may review your medical records.
- Hospital accrediting agencies
- A data safety monitoring board organized to oversee this research.

Some who may receive your protected health information may not have to satisfy the privacy rules and requirements. They, in fact, may share your information with others without your permission.

5. For how long will protected health information about me be used or shared with others?

- There is no scheduled date at which your protected health information that is being used or shared for this research will be destroyed, because research is an ongoing process.

6. Statement of privacy rights:

- You have the right to withdraw your permission for the research doctors and participating DF/HCC entities to use or share your protected health information. We will not be able to withdraw all the information that already has been used or shared with others to carry out related activities such as oversight, or that is needed to ensure quality of the study. To withdraw your permission, you must do so in writing by contacting the researcher listed above in the section: “Whom do I contact if I have questions about the research study?”

- You have the right to request access to your protected health information that is used or shared during this research and that is related to your treatment or payment for your treatment, but you may access this information only after the study is completed. To request this information, please contact the researcher listed above in the section: “Whom do I contact if I have questions about the research study?”

Documentation of Consent

My signature below indicates:

- I have had enough time to read the consent and think about participating in this study;
- I have had all of my questions answered to my satisfaction;
- I am willing to participate in this study;
- I have been told that my participation is voluntary and I can withdraw at any time

______________________________ _________________________
Signature of Participant Date
or Legally Authorized Representative

____
Relationship of Legally Authorized Representative to Participant

DFCI Protocol Number: 14-496	Approved Date (DFCI IRB Approval): 10/13/2016
Date Posted for Use: 11/02/2016	Expiration Date (Invalid for use on or after): 10/13/2017
Version Date (OnCore): 10/24/2016	Content Last Revised with OHRS Review: AM #4

Adult Participants

To be completed by person obtaining consent:

The consent discussion was initiated on ____________________ (date).

Signature of individual obtaining consent: ____

Printed name of above: ____

Date: _______________

☐ A copy of this signed consent form will be given to the participant or legally authorized representative, or, where the participant is a minor, the participant's parent or legal guardian.

For Adult Participants

☐ 1) The participant is an adult and provided consent to participate.

☐ 1a) Participant (or legally authorized representative) is a non-English speaker and signed the translated Short Form in lieu of English consent document:

As someone who understands both English and the language spoken by the participant, I interpreted and/or witnessed, in the participant's language, the researcher's presentation of the English consent form. The participant was given the opportunity to ask questions.

Signature of Interpreter/Witness: ____

Printed Name of Interpreter/Witness: ____

Date: _______________

☐ 1b) Participant is illiterate

The consent form was read to the participant who was given the opportunity to ask questions.

Signature of Witness: ____

Printed Name of Witness: ____

Date: _______________

☐ 2) The participant is an adult who lacks capacity to provide consent and his/her legally authorized representative:

☐ 2a) gave permission for the adult participant to participate

☐ 2b) did not give permission for the adult participant to participate

DFCI Protocol Number: 14-496	Approved Date (DFCI IRB Approval): 10/13/2016
Date Posted for Use: 11/02/2016	Expiration Date (Invalid for use on or after): 10/13/2017
Version Date (OnCore): 10/24/2016	Content Last Revised with OHRS Review: AM #4
